# Rubber Hands Feel Touch, but Not in Blind Individuals

**DOI:** 10.1371/journal.pone.0035912

**Published:** 2012-04-27

**Authors:** Valeria I. Petkova, Hedvig Zetterberg, H. Henrik Ehrsson

**Affiliations:** Brain, Body and Self Laboratory, Department of Neuroscience, Karolinska Institute, Stockholm, Sweden; Royal Holloway, University of London, United Kingdom

## Abstract

Psychology and neuroscience have a long-standing tradition of studying blind individuals to investigate how visual experience shapes perception of the external world. Here, we study how blind people experience their own body by exposing them to a multisensory body illusion: the somatic rubber hand illusion. In this illusion, healthy blindfolded participants experience that they are touching their own right hand with their left index finger, when in fact they are touching a rubber hand with their left index finger while the experimenter touches their right hand in a synchronized manner (Ehrsson et al. 2005). We compared the strength of this illusion in a group of blind individuals (n = 10), all of whom had experienced severe visual impairment or complete blindness from birth, and a group of age-matched blindfolded sighted participants (n = 12). The illusion was quantified subjectively using questionnaires and behaviorally by asking participants to point to the felt location of the right hand. The results showed that the sighted participants experienced a strong illusion, whereas the blind participants experienced no illusion at all, a difference that was evident in both tests employed. A further experiment testing the participants' basic ability to localize the right hand in space without vision (proprioception) revealed no difference between the two groups. Taken together, these results suggest that blind individuals with impaired visual development have a more veridical percept of self-touch and a less flexible and dynamic representation of their own body in space compared to sighted individuals. We speculate that the multisensory brain systems that re-map somatosensory signals onto external reference frames are less developed in blind individuals and therefore do not allow efficient fusion of tactile and proprioceptive signals from the two upper limbs into a single illusory experience of self-touch as in sighted individuals.

## Introduction

The classical question of how blind people experience the external world in terms of space and the shape of objects has attracted much attention from philosophers, psychologists, and neuroscientists through the centuries [1]–[7]. An extensive body of literature describes our substantial knowledge about these questions and information about the effects of visual deprivation on tactile [8] and auditory perception [9]–[11] and on the development of multisensory brain systems [12], [13]. In general, when the brain receives no visual input, this leads not only to changes in the visual system [14], [15] but also to the structural and functional reorganization of brain regions that support other sensory modalities and areas that mediate the integration of information across these modalities [8], [9], [12], [16]. This brain plasticity is associated with behavioral changes that are related to how blind individuals use sensory information from the intact senses to build representations of external space to localize tactile and auditory cues [17], [18]. Visual experience during the early years of life is crucial for the development of multisensory integration mechanisms [12], [13], [19]. Congenitally blind individuals have been reported to exhibit more extensive behavioral [17], [18], [20] and neuronal changes [21]–[23] compared with individuals who had full vision during childhood.

The long-standing tradition of studying how visual experience shapes the perception of the external world contrasts with the lack of studies investigating how blind individuals experience their own body. Given that blind people have an intact somatosensory system, a perfectly reasonable argument could be that they should be able to perceive their own body just as sighted individuals do. However, the experience of our body in space does not depend only on the somatosensory system, as there exists no unique set of peripheral receptors that inform the brain about the location or self-identity of body parts. The spatial experience of our own body must therefore be constructed within the central nervous system by the integration of somatosensory signals (from skin receptors, muscle stretch receptors, joint receptors, etc.) [24]–[30], visual input [31]–[35], auditory signals [36], vestibular cues [37], [38], and stored information related to prior experiences of the body (memory) [31], [39]. If, however, the lack of visual input is sufficient to change the multisensory representations of the spatial properties of objects in the external world, then is it possible that visual deprivation may also influence the multisensory representation of one's own body?

In this study, we used a perceptual illusion – the “somatic” rubber hand illusion [40] – to compare the multisensory representation of the body in blind and sighted individuals. Over the last decade, the rubber hand illusion has become a popular tool to study how we experience ownership of our limbs and the underlying multisensory representation of the body [41]–[47]. In our non-visual version of the rubber hand illusion [40], the investigator moves the blindfolded participant's left index finger so that it touches the knuckle of a rubber right hand; at the same time, the investigator touches the participant's right hand on the knuckle at the corresponding site. After 10 to 15 seconds of these repetitive and synchronous touches [40], most participants start to experience that they are touching their own right hand directly with their left index finger [40]. It has been suggested that this illusion is elicited as a consequence of the brain's attempt to resolve the conflicting tactile and proprioceptive information from the two fingers [40]. The dynamic tactile and proprioceptive information derived from the left index finger correlates with the touches sensed on the right knuckle. This correlation leads to a recalibration of the perceived location of the right hand so that it feels closer to the left hand [40], and consequently, a unitary percept of the left index finger directly touching the participant's right hand is experienced [40]. It has been proposed that this perceptual fusion requires the re-mapping of the touch sensation into a common external reference frame in space near the hands [40], [48], [49]. Consistent with this idea and with data obtained from the original visual version of this illusion [34], the somatic rubber hand illusion is associated with increased activity in the premotor and intraparietal cortices [40], areas that are known to be involved in multisensory integration and encoding of visual and somatic signals in external body-part-centered coordinates [50]–[57].

In this report, we describe what is, to the best of our knowledge, the first investigation of a body illusion in blind individuals. We compared the strength of the somatic rubber hand illusion in a group of blind individuals, all of whom had experienced severe visual impairment or complete blindness from birth, and a group of age-matched blindfolded sighted participants. We quantified the illusion using subjective reports in the form of questionnaires and behavioral data in the form of inaccurate reaching in a pointing task when asked to indicate the position of the right hand. We hypothesized that blind individuals would experience a weaker illusion of touching their own hand because impaired developmental vision should lead to a functional and structural reorganization of multisensory brain circuits, presumably including those that support multisensory body perception. Furthermore, behavioral studies have shown that congenitally blind individuals do not re-map somatosensory signals in external coordinates [17], [18] as sighted individuals do [58], [59]. This suggests a reduced remapping of somatosensory signals to coordinate systems used to encode the space near the body, which are likely to be modulated by vision. Thus, when exposed to the somatic rubber hand illusion, we expected that blind individuals would not perceptually fuse the tactile and proprioceptive signals from their two hands into a single illusory multisensory percept of their own two hands being in direct physical contact as sighted individuals do. However, a weaker illusion in the blind participants could also potentially be explained by their superior ability to localize their hands in space (proprioception) due to sensory compensation and cross-modal plasticity mechanisms [9], [16] within the proprioceptive system. To control for this possibility, we used an established bimanual matching task to assess the two groups' ability to perceive the location of their right hand in a horizontal plane (without vision) [60].

Our results show that the blind participants were less susceptible to the somatic rubber hand illusion compared to the sighted participants, who reported a significant illusion. Importantly, this difference could not be explained in terms of differences in basic proprioceptive ability, as both groups showed similar accuracy in localizing their hand in space. Thus, our results suggest a fundamental difference in the ways that blind and sighted individuals construct a central representation of their own body and identify their own limbs by touch.

## Methods

### Participants

Ten blind (9 female) and twelve sighted (8 female) participants took part in this study. The inclusion criterion for the blind group was complete blindness or severe visual impairment without the ability to see contour or movement. The ability to see light and dark was allowed. The data on the type of visual impairment and number of years without vision are presented in Table 1. Five participants were congenitally blind, and five had been severely visually impaired throughout development and had only been able to see contours or light/dark differences prior to losing their vision completely. All of the sighted volunteers had normal or corrected-to-normal vision. Both the blind and sighted participants were healthy and spoke Swedish or English, and the participants in the two groups were age matched. The mean ages for the blind and sighted participants were 46.8±14.8 years and 43.8±17.2 years, respectively. All of the participants gave their informed consent prior to their participation in these experiments. These studies were approved by the Regional Ethical Review Board of Stockholm.

**Table 1:** Detailed data on the visual impairments observed in the blind group.

Participant (gender, age)	Current vision	Past vision	Blind at what age	Years without vision	Visual impairment
No. 1 (f, 48)	-	Light/dark	2,5	45,5	Retinitis pigmentosa
No. 2 (f, 60)	-	Contours	26	34	Retina
No. 3 (f, 47)	Light/dark	-	Congenitally	47	Retina + optic nerve
No. 4 (f, 66)	-	10%	48	18	Retinitis pigmentosa
No. 5 (f, 23)	-	-	Congenitally	23	Retinitis pigmentosa
No. 6 (f, 44)	Light/dark	-	Congenitally	44	Retinopathy
No. 7 (f, 46)	Light/dark	-	Congenitally	46	Retinopathy
No. 8 (f, 64)	-	2/10	49	15	Retinitis pigmentosa
No. 9 (m, 54)	-	Contours	16	38	Glaucoma + Retina
No. 10 (f, 20)	-	-	Congenitally	20	Retinitis pigmentosa

### General experimental procedures for rubber hand experiments #1 and #2

Before the actual experiment commenced, the participants were verbally informed about the experimental setup. The participants were given detailed instructions about how to place their hands on the table and were informed that they would be touching a rubber hand. In addition, prior to the experiment, the sighted individuals were allowed to look at and touch the rubber hand, and the blind participants were instructed to manually explore the model hand with both hands. Thus, both groups received identical knowledge about the experimental setup and could somatically recognize that they were touching a rubber hand. This setup mirrors the original visual version of the rubber hand illusion, in which the participants had knowledge of the experimental setup and could visually identify the rubber hand.

**Figure 1:**
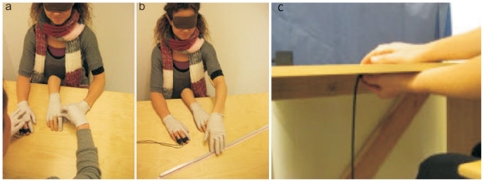
**A**. The setup used to induce the somatic rubber hand illusion. The experimenter moves the blindfolded participant's left index finger so that it touches the rubber right hand while simultaneously touching the corresponding site on the participant's right hand. **B**. The procedure used to register the proprioceptive drift as an objective measure of the illusion (Experiment #2). The participant indicates the felt location of the right index finger by moving the left index finger along a ruler. **C**. The procedure used to measure the basic ability to locate the right index finger in space (proprioception; Experiment #3). The blindfolded participants indicate the felt location of the right index finger resting on the tabletop by moving the left hand under the table to match the positions of both index fingers. The position of the left index finger under the table is registered with a transmitter and the Polhemus Fastrak Magnetic motion-capturing system (see Methods).

During the actual experiments, the participants sat with their arms resting on a table in a pronated position (palms down, Figure 1a). The sighted volunteers were blindfolded for the duration of the experiment. All of the participants were allowed to make small adjustments in the posture of their arms on the tabletop to ensure that they were sitting comfortably and did not have to move their arms during the stimulation periods. A life-sized cosmetic prosthetic male or female right hand (gender-matched) filled with hard plastic was placed on the table between the participant's hands, parallel with the participant's right hand. The distance between the participant's right index finger and the index finger of the rubber hand was always 15 cm.

The participants, the experimenter, and the rubber hand all wore identical plastic surgical gloves to make the tactile surfaces of the hands as similar as possible. The experimenter held the participant's left index finger in a steady grip between the index finger and thumb throughout the entire stroking session, taking care not to change the position of her fingers and to provide the most consistent somatosensory stimulation possible (by not touching any other part of the hand). The participant's lower left arm and hand rested on the table; i.e., he or she did not have to lift the left hand during the stroking sessions. Furthermore, the participant loosely bent all of the fingers of his or her left hand except for the index finger so that the fingers were not in the way of the experimenter's hand or the moving index finger. This configuration ensured that the participants would not accidently touch the rubber hand with any other digits or parts of their left hand.

Using this approach, the experimenter moved the participant's left index finger so that it stroked the index finger of the rubber hand. She also stroked the participant's right index finger either in synchrony (experimental condition) or temporal asynchrony (control condition) (Figure 1a). Each stroke was 3–5 cm long and applied in proximal-to-distal or distal-to-proximal directions, always passing at least one joint (metacarpophalangeal or proximal interphalangeal), for approximately one second. The stroke direction alternated in a quasi-randomized way, and the participant's and the experimenter's index fingers were lifted after each stroke. The period between the strokes was approximately one second. In the asynchronous condition, the strokes were applied in an alternating fashion so that when the participant's left index finger touched the rubber hand, no tactile stimulation was delivered to the participant's right index finger, and vice versa. Each stroking session lasted 60 seconds. The experimenter was trained extensively during pilot testing to deliver these touches as consistently as possible with respect to synchronicity, force, duration and frequency.

### Experimental design

#### Experiment #1: Questionnaire data

**Figure 2:**
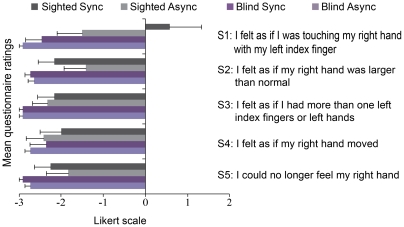
The results from the questionnaire (Experiment #1). The blind participants firmly denied the illusion statement (Statement 1; S1) under both synchronous and asynchronous conditions (for further details, see Table 2). The participants also denied all of the control statements (S2–S5). The sighted participants affirmed the illusion on average (S1), but only in the synchronous condition (dark gray). The percentage of participants who affirmed the illusion and the rating of S1 in the synchronous condition were significant between the groups (*p*<0.05). For details about the statistical analysis, see the Results section.

The participants were exposed to two stroking sessions of synchronous or asynchronous stimulation, each of which lasted 60 seconds. At the end of each session, the participants were asked to complete a verbally administered questionnaire in which they had to rate their experience of five possible perceptual effects using a seven-point Likert scale, ranging from ‘−3’ (I disagree very strongly) to ‘+3’ (I agree very strongly), with ‘0’ indicating “I am uncertain.” One statement was designed to capture the illusory experience of touching one's own hand, and the other four served as controls for suggestibility and task compliance (Figure 2).

#### Experiment #2: Proprioceptive drift

This experiment consisted of 6 stroking sessions (3 synchronous and 3 asynchronous), each lasting 60 seconds. Immediately before and after the sessions, the participants were required to point with their left index finger towards their right index finger (Fig. 1b). A 70-cm-long scale and a measuring tape were placed on the table 10 cm in front of the participant's right hand. The scale was oriented at a 90-degree angle with respect to the participant's right hand. The participant was asked to place his or her left index finger on the scale and slide it in a single brisk movement towards the felt position of the right index finger. The pointing error, i.e., the drift of the felt position of the right hand from its actual position, was calculated as the difference in pointing before and after the stroking sessions. The crucial test of the illusion was to compare the pointing error during the synchronous and asynchronous conditions and to look for significantly greater drift towards the rubber hand in the synchronous condition [40]. This proprioceptive drift measure has previously been used as an index of the rubber hand illusion [41] (for a recent critical review of the limitations of the drift measure, see [61]). Between each session, the participant was given a short break of 30 seconds and was asked to move his or her right arm and hand. The rationale for this break was to eliminate any potential illusory perception and to exclude any possible carry-over effects.

#### Experiment #3: Finger localization task to test proprioception

In this experiment, the participants were seated at a table where the right hand was placed on top of the table and the left hand below the tabletop. Small pieces of cardboard, each the size of a fingertip, were glued onto the table top at the following *x* and *y* coordinates, where the origin of the coordinate system was the upper left corner of the table: (i) 30.00 cm×17.00 cm; (ii) 14.00 cm×31.50 cm; (iii) 26.00 cm×51.00 cm. The participants were required to place the tip of their right index finger on one of the three marked locations and to match its position as accurately as possible with the tip of their left index finger beneath the table top (Figure 1c). The participants were allowed to take their time to find the correct position. Whenever they verbally reported that the two index fingers were placed directly on top of each other, the position of the receiver at the tip of the left index finger was recorded. Between every trial, the participants placed their hands in the starting positions at the corners of the table, with the left hand positioned under the table and the right hand placed on top of the table. The participants were asked not to move their body with respect to the chair, which was also monitored by the experimenter.

Proprioceptive accuracy was measured fifteen times at each location; the order of the trials was quasi-randomized and was the same for all participants. The measurements were performed with the Polhemus Fastrak Magnetic motion-capturing system (http://www.polhemus.com/). This system consists of a transmitter, which was fixed at the upper left corner of the table, i.e., at the origin of the coordinate system. The motion-capturing system transmits a magnetic field to register the position of the receiver, which was positioned on the tip of the participant's left index finger. The distance from the transmitter to the receiver was measured in centimeters along the *x*– and *y*-axes of the coordinate system. The accuracy of the receiver in the workspace was 0.8 mm.

### Order of experiments and post-experiment interviews

Experiment #2 (proprioceptive drift) was always conducted before Experiment #1 (questionnaire) to prevent the participants from guessing the specific purposes of the objective measures on the basis of the questionnaire statements in Experiment #1. Experiment #3, the bimanual finger localization task, was always conducted last, as it required a different setup than the two rubber hand illusion experiments. The blind and sighted groups were tested in the same run of experiments, using an identical experimental setup with the same experimenter.

At the end of the last experiment, we interviewed the participants about their subjective experiences. The participants were asked to describe their experiences during the testing conditions, their experiences with their hands, and how and where they had sensed the touches. In addition, the participants were debriefed about the illusion and the most common illusory percepts associated with it, and were asked to compare those to their own experiences.

### Statistical analysis

We used two hypothesis-driven statistical analyses. First, we tested for a significant difference in the strength of the illusion *between* the two groups. To test this, the two groups were compared in a mixed analysis of variance (ANOVA) with group (blind vs. sighted) as a between-subjects factor (experiment #2). In experiment #1, the data were not normally distributed (e.g., most blind individuals gave a rating of −3 on the illusion statement). Therefore, we used non-parametric tests to directly compare the rated strength of the illusion between the groups. Second, we tested for the presence of the illusion *within* each group in a planned comparison approach using *t*-tests (or equivalent non-parametric tests). In all tests, we defined a significant *p* value as *p*<0.05 (two-tailed).

## Results

### Experiment #1: Questionnaire data

The questionnaire data showed that 67% (8 out of 12) of the sighted participants affirmed the illusion of touching their own hand (i.e., score ≥+1 on the illusion statement S1), compared with only 10% (1 out of 10) of the blind participants (Figure 2 and Table 2). The difference in the number of affirmative scores between the groups was significant (*p* = 0.011, Fisher test). Thus, significantly more sighted than blind individuals affirmed that they experienced the illusion. An inspection of the responses to the control items (S2–S5) showed that both of the groups strongly rejected the control items, which suggests that their responses to the illusion statement were truthful.

**Table 2:** Descriptive data on individual participants' scores on the illusion statement (S1, see Figure 2).

Statement S1 (illusion)	Sighted (n = 12)		Blind (n = 10)	
7-point Likert scale	No. of subjectsSync (Async)	Percentage Sync (Async)	No. of subjects Sync (Async)	Percentage Sync (Async)
+3	4 (1)	33% (8%)	0 (0)	0 (0)
+2	2 (0)	17% (0%)	0 (0)	0 (0)
+1	2 (2)	17% (17%)	1(0)	10 (0)
0	0 (0)	0% (0%)	0 (0)	0 (0)
−1	0 (1)	0% (8%)	0 (0)	0 (0)
−2	1 (2)	8% (17%)	0 (1)	0 (10)
−3	3 (6)	25% (50%)	9 (10)	90 (90)

Next, we directly compared the rated strength of the illusion between the two groups. The sighted group showed statistically higher rating scores than the blind group on the illusion statement (S1) in the synchronous condition (*p* = 0.009, two-tailed independent samples Mann-Whitney *U* test). We observed no significant differences in the scores on the S1 statement between the groups in the asynchronous condition (*p* = 0.093, two-tailed independent samples Mann-Whitney *U* test). Moreover, no between-group differences in the ratings on the 4 control statements were observed in either the synchronous or asynchronous conditions (S2–S5) (*p*>0.1 two-tailed independent samples Mann-Whitney *U* test) (Figure 2). Thus, the sighted individuals reported perceiving a stronger illusion than the blind participants did.

We also compared the strength of the illusion (as rated on S1) between the synchronous and asynchronous conditions within each group. Statistically, this effect of the illusion (synchronous vs. asynchronous condition) was present only in the sighted participants (p = 0.048, two-tailed Wilcoxon rank sum test) and was not significant for the blind subjects (p = 0.18, two-tailed Wilcoxon rank sum test) (Figure 2).

As described in Table 2, 9 out of the 10 blind participants gave the lowest possible rating for their experience of the illusion (−3 on statement S1). The only blind person who reported experiencing the illusion was congenitally blind. She affirmed the illusion in the questionnaire (+1; synchronous condition), but also affirmed sensing the hand moving, which was one of the control statements (+1; synchronous condition). It is noteworthy that this person had undergone training with her parents during her childhood in which she was taught how to interact with objects in her environment in a relatively free and unrestricted way. The overall pattern of her behavior seemed to differ from the behavior of the rest of the blind individuals in that she exhibited more proactive and exploratory behavior.

### Experiment #2: Proprioceptive drift

To complement the questionnaire results, we obtained a well-established objective measure of the rubber hand illusion (Experiments #2). We asked the participants to point with their left index finger towards the felt position of their right index finger [40]. The pre- to post-test difference measures the proprioceptive error induced by the experimental condition. Importantly, a greater proprioceptive drift towards the rubber hand in the synchronous condition compared to the asynchronous condition can be used as an objective measure of the classical [43] and non-visual versions of the rubber hand illusion [40].

A direct comparison of the illusion-specific drift between the groups revealed that the sighted participants displayed a greater drift towards the rubber hand when subtracting the asynchronous responses from the synchronous responses compared to the blind group (significant interaction between group (blind vs. control) and timing of stimulation (synchronous vs. asynchronous); *p*<0.001, F = 18.254, mixed ANOVA).

**Figure 3:**
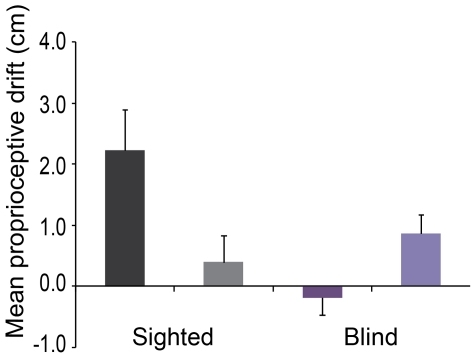
The results from the proprioceptive drift test, which served as an objective measure of the illusion (Experiment #2). As expected, the sighted individuals showed a significantly greater drift in the perceived location of their right hand towards the location of the rubber hand in the synchronous condition than in the asynchronous condition (*p*<0.05). This illusion-specific drift towards the rubber hand was absent in the blind group, which did not show any drift in the synchronous condition (mixed ANOVA showed a significant difference between groups, *p*<0.05). For details about the statistical analysis, see the Results section. Synchronous conditions are color-coded dark gray (sighted) and dark purple (blind), and the asynchronous conditions light gray (sighted) and light purple (blind).

Planned comparisons showed that the sighted participants displayed a significantly greater (*p* = 0.006, two-tailed paired *t*-test) proprioceptive drift towards the rubber hand in the synchronous condition (2.2 cm±2.3 cm; mean±SD) compared to the asynchronous condition (0.4 cm±1.5 cm; mean ± SD). In contrast, the blind participants did not display this illusion-specific proprioceptive drift response. Instead, the blind participants showed almost no proprioceptive drift in the synchronous condition (0.1 cm±0.8 cm; mean ± SD), and surprisingly, a significantly larger (*p* = 0.017, two-tailed paired *t*-test) drift towards the rubber hand in the asynchronous condition (0.8 cm±1.0 cm; mean ± SD) (Figure 3). The unexpected drift observed in the asynchronous condition was of a comparable magnitude to the drift demonstrated by the sighted participants after the asynchronous condition (*p* = 0.416, two-tailed unpaired *t*-test). It has been demonstrated that small changes in proprioceptive drift can also occur in the absence of changes in illusory ownership [61]. Therefore, we believe that the observed proprioceptive drift after asynchronous visuo-tactile stimulation in the blind group most likely has no behavioral significance to the illusion under investigation.

Finally, we examined if the pointing error before the experimental manipulation was similar between the blind and sighted participants. Importantly, the pointing error in the pretest condition did not differ significantly between the two groups (*p* = 0.808, two-tailed unpaired *t*-test). This result suggests that the sighted and blind individuals were equally capable of localizing their hands in the setup prior to synchronous or asynchronous visuo-tactile stimulation. Hence, the differences we observed after the experimental conditions were likely related to differences in illusion susceptibility rather than to differences in the ability to perform the pointing task or in the capacity to perceive the location of the right hand.

### Experiment #3: Finger localization task to test proprioception

**Figure 4:**
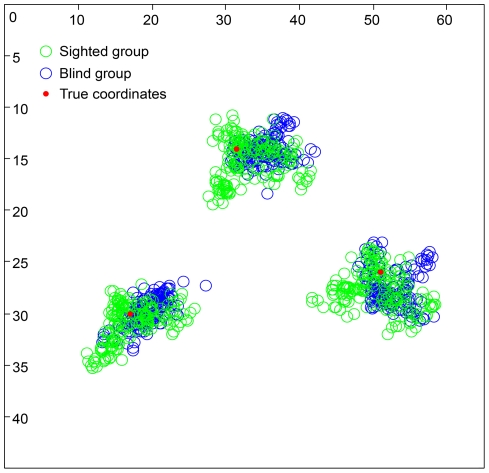
The results from the finger localization task that tested the participants' basic proprioceptive ability (Experiment #3). The graph illustrates the perceived location of the right index finger on the table, as indicated by the participant moving his or her left index finger under the table. The data from the two different groups are indicated by the different colored circles (sighted: green circles; blind: blue circles). The red circles indicate the actual location of the target right index finger on the table. The values on the *x*- and *y*-axes refer to distances from the top left corner of the table in cm. There were no significant differences between the finger-pointing responses in the two groups (see the Results section for additional details about the analysis).

In this experiment, we tested the two groups on their basic ability to localize their right hand by relying on proprioception alone [60]. We found no difference in the proprioceptive accuracy between the blind and sighted participants (*p* = 0.41, mixed ANOVA) (Figure 4). Consistent with previously reported results [60], we also found a significant main effect on target location. We found that the proprioceptive accuracy was higher for locations close to the body than for locations far away from the body (*p* = 0.005, mixed ANOVA). There was no difference in this pattern between the blind and sighted participants, as indicated by the non-significant interaction between target location and group (*p* = 0.16, mixed ANOVA).

## Discussion

In this study, we found that blind and blindfolded sighted participants differed profoundly in their ability to experience a non-visual version of the rubber hand illusion. Whereas the vast majority of blind individuals appeared “immune” to the illusion, sighted participants perceived a strong illusion of touching their own hand when they were in fact touching a rubber hand, consistent with previously published results [40] (and consistent with versions of this illusion where sighted participants touch the rubber hand with a paintbrush; see [62], [63]). This difference between the groups in their capacity to experience the illusion was supported by converging data derived from the results of the questionnaire rating scales and the manually reported proprioceptive drift. More specifically, between-group comparisons revealed that the sighted participants gave significantly higher affirmative ratings of the illusion (*p*<0.05) and displayed significantly greater proprioceptive drift towards the rubber hand in the synchronous condition compared to the asynchronous control (*p*<0.05). Furthermore, the blind participants showed a remarkably strong denial of the illusion, with 9 out of 10 participants giving the lowest rating (−3) of the illusion statement in the questionnaires. Anecdotally, we found that most of the blind individuals maintained this strong rejection of even a very weak illusion response in the informal post-experiment interviews. When asked to comment on the illusion statement, several of the blind participants remarked that it was “totally absurd” or that they “could not even imagine the illusion.” Taken together, these results suggest a fundamental difference in the way that tactile and proprioceptive information from the two hands is integrated at the central level to construct a unitary experience of one's own two hands in direct contact. Our interpretation is that blind individuals do not re-map somatic signals to an external reference frame in near-personal space as sighted people do, and therefore do not perceptually fuse the somatic signals from their two hands into a single event of self-touch (see further discussion below).

The fact that blind individuals were not susceptible to the somatic rubber hand illusion could not be explained simply by their enhanced ability to localize their hands in space. The blind and blindfolded sighted individuals showed no significant differences in this ability when directly tested in the bimanual matching finger localization task (experiment #3). Furthermore, in both groups, we reproduced a previously reported spatial pattern of precision where hand positions closer to the shoulder were localized more precisely than those located further away [60]. In addition, we observed no significant differences in “baseline” pre-stimulation proprioceptive drift measurements between the groups (experiment #2). Thus, although extraordinary sensory abilities have been reported in the auditory and tactile domains in blind individuals [3], [11], [64], [65], our results do not support the assumption that such effects are present in the proprioceptive system for hands. Therefore, the differences in the inducibility of the illusion between the groups can be explained better by differences in how information from the different sensory channels is integrated at the central level (multisensory integration) than by differences in the unimodal processing of proprioceptive signals. It is relevant here to point out that most of our blind participants had received orientation and mobility training as young children, and it is known that such training facilitates proprioceptive-spatial discriminative ability, which could explain why they performed at the same level as the sighted participants in our proprioceptive tests [66].

The elicitation of the somatic rubber hand illusion is a direct consequence of the integration of tactile and proprioceptive information from the two hands [40]. Why would this process be different for blind and sighted individuals? We can propose two alternative, non-mutually exclusive explanations that are both consistent with our data. First, blind individuals might not encode somatosensory signals into external reference frames and therefore not fuse the correlated tactile information from the two fingers as sighted individuals do. Previous experiments have shown that in sighted individuals, tactile stimuli are automatically re-mapped to external coordinates [58], [59], [67] and that multisensory parietal [50], [53], [55], [58], [68] and premotor areas [50], [53], [69] are likely to be involved in this process. In contrast, congenitally blind individuals do not seem to employ such re-mapping of tactile stimuli [17], [18]. In tasks that employ temporal order judgments of tactile stimuli that are applied to the two hands in rapid succession [59], [67], [70], it has been shown that congenitally blind individuals perform better than sighted individuals and late blinds when the hands are crossed over the midline, suggesting that they rely more on anatomically based information rather than a common external coordinate system for action control and perception [17], [18]. Presumably, congenitally blind individuals show reduced remapping of somatosensory signals to all external coordinate systems that are likely to be modulated by visual input, including body-part-centered coordinates. Because the rubber hand illusion depends on the integration of multisensory information in external body part-centered reference frames [34], [40], [52], [71], [72], a reduced general ability to employ tactile re-mapping to external coordinate systems in the blind could explain their inability to experience the illusion.

An alternative interpretation is that touch and proprioception are weighted differently in blind and sighted individuals. In the classic model of multisensory integration, the integration process is conceptualized as resulting from a linear summation of signals obtained from two or more modalities, where each signal is weighted according to its reliability [73]. This idea is conserved in contemporary probabilistic models of multisensory integration, albeit with the caveat that the “weighing” can vary dynamically depending on the context [74]–[76]. In our paradigm, a greater weight given to proprioceptive information from the hands and arms should work against the illusion, as the spatial discrepancy between the two hands would be maintained and the correlated tactile signals “ignored.” In contrast, a greater weighing of touch should work in favor of the illusion, as it is the detection of the spatially and temporally congruent tactile signals that drives the illusion. As discussed above, the two groups revealed no difference in their basic ability to localize their right index finger in space, which speaks against possible differences in the weighing of proprioception. Although we did not measure tactile acuity in this study, there is an ample amount of published evidence that suggests that blind individuals have a superior spatial and temporal tactile discriminative ability (see below). Thus, if anything, one would expect greater weighing of touch in the blind group, which presumably would have intensified the illusion. Our findings are better explained by a model in which the lack of illusory self-touch in blind individuals is attributed to a reduced ability to integrate tactile and proprioceptive information from the two hands into a common external reference frame in the peripersonal space [40].

After this analysis of multisensory models, it is important to also carefully consider the possibility that the lack of an illusion in the blind could be simply accounted for by superior tactile processing [64], [77], [78] (i.e., by differences in unisensory tactile processing). All of our blind participants were Braille readers, and it is known that Braille readers show greater tactile acuity in their fingertips than individuals who do not read Braille [78]. Thus, it could be that blind individuals are more sensitive to incongruence in the texture between the rubber hand and their own hand. To quote an anonymous reviewer, “the rubber hand might feel too fake to be part of their own body.” Although we cannot exclude this possibility, we are nevertheless of the opinion that this is not a likely explanation because of three relevant aspects of tactile processing that could conceivably affect the rubber hand illusion: (i) perception of roughness or texture (also referred to as microgeometry of the object); (ii) the ability to perceive surface curvature that underlies shape perception (i.e., the macrogeometric properties of the object); and (iii) the temporal perception of the relative timing of the tactile stimuli. With respect to the first point, we know that the rubber hand illusion is insensitive to differences in texture, and that it can be induced under conditions of substantially greater mismatch in texture than in our current experiments [63], [79]. Indeed, during the somatic rubber hand illusion, participants typically perceived (and spontaneously remarked on) the unnatural texture, temperature and stiffness of the rubber hand, but, nevertheless, were able to incorporate these tactile percepts into their experience of touching their own hand. Furthermore, all of the participants in our study wore plastic surgical gloves on both hands, which reduced the perceived differences in texture. Second, both groups received identical prior knowledge that they were touching a rubber hand and were able to recognize the shape of the rubber finger. Although we know that the rubber hand illusion breaks down when the participant sees or touches an object that does not resemble a human hand at all (like a block of wood [39] or a rectangular dish brush [40]), it is unlikely that the putative better shape perception of blind individuals would change their object perception so dramatically that they would no longer perceive it as an object shaped like a finger. In addition, it is important to note that the empirical support for the assumption that blind individuals have a superior ability to haptically recognize the shape of objects is mixed (as reviewed by [80]). Third, although experimental data have shown that the congenitally blind have a superior capacity to detect the relative timing of tactile stimuli delivered to the two hands (for example, 23 ms for the congenitally blind compared to 47 ms in sighted individuals [17]), we think that it is unlikely that this difference can explain our results. First, great care was taken by a highly trained experimenter to administer the touches as synchronously as possible. Second, the touch stimuli involved stroking the finger along a continuous 2-to3-cm path length. This mode of stimulation provides a strong spatial cue for congruence that would not depend on the fine-grained temporal resolution of tactile perception. Third, the classical and somatic rubber hand illusions are quite robust to subtle violations in synchrony (anecdotal observations), and the classical visual rubber hand illusion can be maintained with inter-sensory delays as long as 300 ms under certain experimental conditions [81]. Finally, none of the blind participants remarked that the touches were asynchronous (in the illusion condition) when they were asked in the post-experiment interviews to speculate about why the illusion did not work.

What do these findings tell us about the rubber hand illusion as a model system to study body ownership? It has been proposed that the rubber hand illusion can be explained in terms of the integration of visual, tactile and proprioceptive information [34], [41], [43]. This idea has been extended to models where perceived changes in ownership during the illusion are linked to the integration of multisensory information into body-part-centered reference frames [34], [48], [49]. Similarly, models that maintain the feeling of ownership as a higher-order cognitive process distinct from multisensory integration mechanisms nevertheless emphasize that “recalibration of visual and tactile coordinates and the referral of touch” are mandatory early-stage components of the embodiment process [82]. Thus, under the assumption that the lack of normal visual experience during childhood leads to a reduced multisensory representation of the upper limbs in peripersonal space, our current finding that blind participants appear “immune” to the somatic rubber hand illusion supports multisensory models of body ownership [48], [49], [82].

Our results have left one important question unanswered: how do blind individuals experience ownership of their body? In psychology, there is a long-standing tradition of studying illusions to learn more about the mechanisms that mediate normal perception. Thus, the experience of the rubber hand illusion in the sighted group informs us about the mechanisms of ownership in sighted individuals. However, the lack of an illusion in the blind group does not directly inform us about the mechanisms that produce ownership in blind individuals. Although a multisensory integration mechanism, albeit in altered form (e.g., in terms of reference frames or ‘weighing,’ as discussed above), may still be the most likely candidate mechanism for body ownership, more studies are needed to clarify the exact nature of such self-attribution processes in the blind. For example, future studies should test if body illusions that presumably do not depend on external coordinates, such as tendon vibration illusions [83], can be elicited in blind participants. It would also be very interesting to examine the potential role of auditory cues in the self-identification of limbs, which might be more important for blind individuals, given that auditory stimuli are also represented in the body-part-centered reference frames in space near the body [36], [57], [84].

A limitation of the design of this study is that we did not conduct a direct comparison between congenitally blind and late-blind individuals. Thus, we were not able to directly test the role of early critical periods during development in the elicitation of the rubber hand illusion [16]. Our blind participants were either congenitally blind (n = 5) or had severe visual impairments in childhood before the onset of adult blindness (n = 5) (see Table 1). It should be noted that there appeared to be no systematic difference in the questionnaire scores or proprioceptive drift responses between the congenitally blind participants and those who had severely impaired vision from birth when we inspected the data using a descriptive approach. Because all of our blind participants demonstrated severely disturbed developmental vision (no functional vision), we can infer substantial structural and functional plasticity in their multisensory brain systems [12], [13], including a likely reduced ability to employ an external reference frame for localizing somatosensory signals [17], [18].

In conclusion, our results suggest that blindness with disturbed developmental vision precludes the elicitation of the somatic rubber hand illusion. This finding suggests the existence of fundamental differences in central body representation between blind and sighted individuals. Specifically, our results suggest that blind individuals with impaired developmental vision have a less dynamic multisensory representation of their own limbs. We can only speculate about the consequences of this “less plastic” body representation for behavioral and cognitive functions. However, it is worth reflecting on a potential link to the characteristic cautious, slow and less explorative movement pattern of the blind [85]. If we conceptualize near-personal space as a special zone that surrounds the body, which is used not only to encode multisensory limb positions in external coordinates but also to facilitate object-directed and defensive actions [51], [52], [86]–[90], then a reduced near-personal space representation in the blind could explain both their inability to experience the somatic rubber hand illusion and their overall reduced mobility and cautious explorative movements.
